# Anti-HMGB1 antibody is a potential characteristic autoantibody for Sjögren's syndrome

**DOI:** 10.1038/s41598-022-10007-3

**Published:** 2022-04-11

**Authors:** Mingkun Chen, Yi Zhou, Miao Xue, Rongrong Zhu, Liling Jing, Liling Lin, Chengwen He, Yanghua Qin

**Affiliations:** 1 Department of Laboratory Medicine, Changhai Hospital, SMMU, Shanghai, 200433 China; 2 School of Basic Medical Sciences, Shanghai University of Traditional Chinese Medicine, Shanghai, 201203 China; 3 Department of Infectious Diseases, Changhai Hospital, SMMU, Room 404, No. 9 Building, 168 Changhai Road, Shanghai, 200433 China; 4 Department of Laboratory Medicine, Changhai Hospital, SMMU, Room 714, No. 5 Building, 168 Changhai Road, Shanghai, 200433 China

**Keywords:** Diagnostic markers, Rheumatic diseases

## Abstract

Sjögren's syndrome (SS) is a common chronic inflammatory autoimmune disease that affects about 0.33–0.77% population in China. The positive for antinuclear antibodies (ANA) is one of the key features of SS, which shows a nuclear fine speckled (AC-4) pattern in an indirect immunofluorescent antibody test (IIFT). About 70% of ANA-positive SS patients have detectable anti-SS-A and/or SS-B antibodies, which indicates that other autoantibodies may present in SS patients. The anti-HMGB1 antibodies in 93 SS patients and 96 healthy controls were investigated with in-house developed ELISA and immunoblotting, and the locations of HMGB1 and fluorescent pattern of anti-HMGB1 antibody were investigated with IIFT. The contribution of anti-HMGB1 antibody in ANA-IF was evaluated with Cas9-induce HMGB1 knockout B16 cells. The anti-HMGB1 antibody level is higher in SS patients (9.96 ± 5.55 RU/ml) than in healthy controls (4.9 ± 1.4 RU/ml). With ROC curve analysis, when taking 8 RU/ml as the cutoff value, the sensitivity, specificity, and the area under the curve were 64.5%, 96.9%, and 0.83, respectively. A total of 18 patients (20.7%) with nuclear fine speckled (AC-4) pattern in ANA-IF test were anti-HMGB1 antibody positive only. With commercial antibody, anti-HMGB1 antibody showed the same nuclear fine speckled (AC-4) pattern. The serum from ANA-IF (+), SS-A (−), and SS-B (−) SS patients showed nuclear fine speckled (AC-4) pattern in wildtype B16 cells, but no fluorescence in HMGB1 knockout B16 cells. Anti-HMGB1 antibody may be one of the characteristic autoantibodies of SS in addition to anti-SS-A and SS-B. The detection of anti-HMGB1 antibody can provide more laboratory evidence for clinical diagnosis of SS.

## Introduction

Sjögren's syndrome (SS) is a chronic inflammatory autoimmune disease characterized by the invasion of exocrine glands. Its clinical manifestations include the symptoms caused by dryness of the glands and the symptoms caused by the involvement of the extraglandular system^1,2^. In China, the incidence of SS is between 0.33 and 0.77%, which is the second-highest prevalence of autoimmune disease after rheumatoid arthritis (RA), and the male to female incidence rate is about 1:9 to 1:19^3^. The pathogenesis of SS has not been fully elucidated. The positive for antinuclear antibodies (ANA) is one of the key features of SS, which shows a nuclear fine speckled (AC-4) pattern in indirect immunofluorescent antibody test (IIFT). In the clinical laboratory, 70% of the SS patients can show anti-SS-A antibody, and about 45% of them can show anti-SS-B antibody. Nucleoprotein and nucleic acid are important sources of autoantigens. At present, it is known that SS-A is composed of protein antigens of 52 kDa and 60 kDa combined with cytoplasmic RNA species, and SS-B is composed of a 48-kDa protein combined with RNA species. Partial confirmed SS patients show positive for ANA in IIFT, while negative for SS-A/SS-B in antibody test; which indicates SS patients have other autoantibodies. According to other literatures and our preliminary researches^4–6^, the high mobility group box 1 (HMGB1) played a key role in inflammation-related immune regulation, and other studies have shown that anti-HMGB1 autoantibody can be detected in a variety of autoimmune diseases. In this article, the possibility of HMGB1 being one of SS related autoantigens was systematically investigated.

## Materials and methods

### Sources of specimen

A total of 93 patients diagnosed as SS according to the SS diagnostic criteria of the Chinese Association of Rheumatology (CAR) were included in the SS group^7^, and another 96 healthy persons were recruited into the control group. The SS diagnostic criteria of the CAR is comparable with that of the ACR-EULAR; the main difference is the CAR includes the items of salivary gland involvement^8^. The demographical data, e.g., age and sex, were collected. The serum samples were collected and stored at − 80 °C. This study used the remaining samples from the clinical laboratory of Changhai Hospital therefore it was exempt informed consent. The study was reviewed and approved by the Medical Ethics Committee of Changhai Hospital, and all the procedures followed the guidelines of the Helsinki Declaration during the study.

### In-house developed ELISA test of anti-HMGB1 antibody

Referencing other studies^9^, the procedure of serum anti-HMGB1 antibody test was as follows: Maxisorp polystyrene 96-wells plates were coated with 50 μl per well of rHMGB1 (R&D Systems, Minneapolis, USA) at 1 μg/ml in PBS and incubated overnight at 4 °C. After one wash, plates were blocked with Blocker Casein (Thermo, Rockford, USA) for 1 h. Serum samples, diluted 1:50 with the sample buffer, were added in duplicate (100 μl/well) and incubated for 2 h at room temperature. After five washes, 100 μl HRP-conjugated rabbit anti-human IgG (Euroimmun, Lubeck, Germany) was added to each well and incubated for 30 min at room temperature. After washing, bound antibodies were detected using 3,3′,5,5′-tetramethylbenzidine dihydrochloride/hydrogen peroxide (TMB/H_2_O_2_). The reaction was stopped with 0.5 M sulphuric acid and the absorbance was measured at 450 nm using a microplate-spectrophotometer (Thermo MK3, Rockford, USA). Anti-HMGB1 antibody levels were expressed in relative units.

### Indirect immunofluorescence test: IIFT

There were three different IIFTs in this study. Antinuclear antibody immunofluorescence kit (Euroimmun, Lubeck, Germany. Item No. FC 1510) was used to perform the ANA-IF test, and the test took fixed HEp-2 cells and monkey liver slices as the detection matrix. All procedures followed the manufacturers’ instructions.

Anti-HMGB1 antibody fluorescence pattern study used the same fixed HEp-2 cells and monkey liver slices as those in the ANA-IF test. The anti-HMGB1 antibody (AB79823, Abcam) and isotype control (AB172730, Abcam) diluted at 1:100 were used as the primary antibodies. The secondary antibody was alex488-goat-anti-rabbit IgG antibody (AB150077, Abcam) diluted at 1:200. The detection procedure was the same as above.

In order to study the effect of HMGB1 knockout on the serum ANA-IF pattern of SS patients, mouse melanoma cell lines (Wild type B16 and HMGB1 knockout type B16^HMGB1−^) fixed on slides were used as the matrix to detect the fluorescence patterns of antinuclear antibodies in SS patients. The secondary antibody used the FITC-goat anti-human IgG antibody (Euroimmun, Lubeck, Germany). The detection procedure was the same as above.

The ANA-IF patterns were carefully reviewed by two laboratory technicians (Miao Xue and Mingkun Chen) according to the International Consensus on Antinuclear Antibody Patterns^9^.

### Immunoblotting test

Anti-SS-A and anti-SS-B antibodies were detected with Euroline ANA profile kits (Euroimmun, Lubeck, Germany) on an automatic EUROBlotone instrument according to manufacturer’s protocol. Anti-HMGB1 antibodies were further confirmed by an in-house developed immunoblotting: Nitrocellulose membrane was coated with three bands of different amounts of rHMGB1 protein (0.001, 0.01, and 0.1 μg) as well as a control band. The strips were incubated with 15 μl serum for 2 h at room temperature. After 3 washes, ALP-conjugated goat anti-human IgG (Euroimmun, Lubeck, Germany) was added to each lane and incubated for 30 min at room temperature. After washing, bound antibodies were detected using nitro blue tetrazolium/5-bromo-4-chloro-3-indolyl phosphate (NBT/BCIP). The bands in the strip were detected with a scanner and analyzed with EUROLineScan.

### Statistical analysis

All data were expressed as $\overline{{\text{x}}}$ ± SD. Student *t* test was used for the comparison between two groups. The ROC curve was used to evaluate the diagnostic efficacy of anti-HMGB1 antibodies in SS with Graphpad^®^ Prism 6. *P* value less than 0.05 indicates statistical significance.

### Ethics approval and consent to participate

This study was approved by the Ethics Committee of Changhai Hospital (81471606). The need for patient consent was waived due to this study used the remaining samples from the clinical laboratory of Changhai Hospital and the absence of personally identifiable data in the report.

## Results

### Anti-HMGB1 autobodies in the serum of SS patients were significantly increased

**Figure 1:**
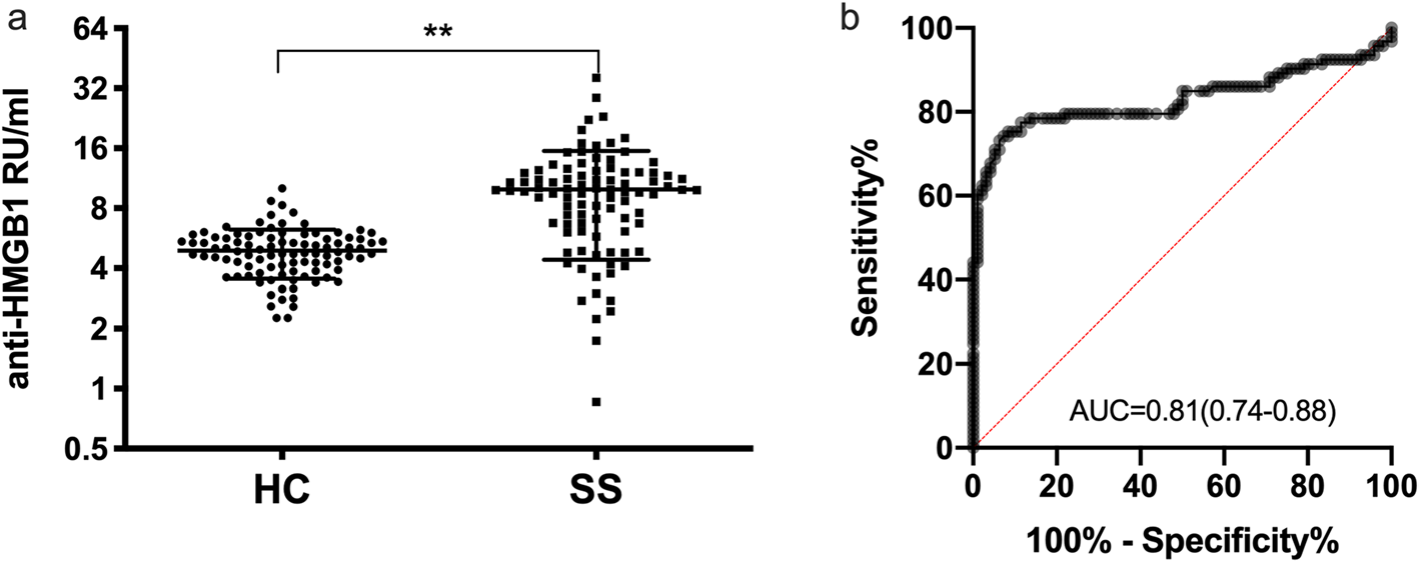
Serum anti-HMGB1 antibody in SS patients. (**a**) ELISA results showed that the anti-HMGB1 antibody were 4.9 ± 1.4 RU/ml and 9.96 ± 5.55 RU/ml in healthy controls and SS patients, respectively. The comparison between the healthy controls and SS patients was tested by Student’s *t* test. ** represents *P* < 0.01. (**b**) ROC curve analysis was performed to calculate the optimal cutoff value of anti-HMGB1 antibodies in the diagnosis of SS. When taking 8 RU/ml as the cut-off value, and the sensitivity and specificity were 64.5% and 96.9% respectively, and the AUC was 0.83 (95% confidence interval: 0.76–0.90).

The SS patients aged 23–80 (median, 52 years old) (Table 1). Among them, 66.7% tested positive for SS-A antibodies, while 40.9% tested positive for SS-B antibodies. The results of anti-HMGB1 ELISA showed the content of anti-HMGB1 autobodies in SS patients (9.96 ± 5.55 RU/ml) were significantly higher than those of the control group (4.9 ± 1.4 RU/ml) (Fig. 1a, Suppl Fig. 1). In ROC curve analysis, when taking 8 RU/ml as the cutoff value, the sensitivity and specificity were 64.5% and 96.9% respectively, and the area under the curve was 0.83 (95% confidence interval: 0.76–0.90) (Fig. 1b).

**Table 1:** Clinical characteristics and serum anti-HMGB1 in SS and healthy controls.

	SS	Healthy control
Sex (M/F)	5/88	53/43
Age	52 (23–80)	52 (15–84)
Anti-SS-A	67.7% (63/93)	–
Anti-SS-B	39.8% (37/93)	–
Anti-HMGB1	9.96 ± 5.55	4.9 ± 1.4

### Detection of related autoantibodies in SS patients with ANA-IF positive

**Figure 2:**
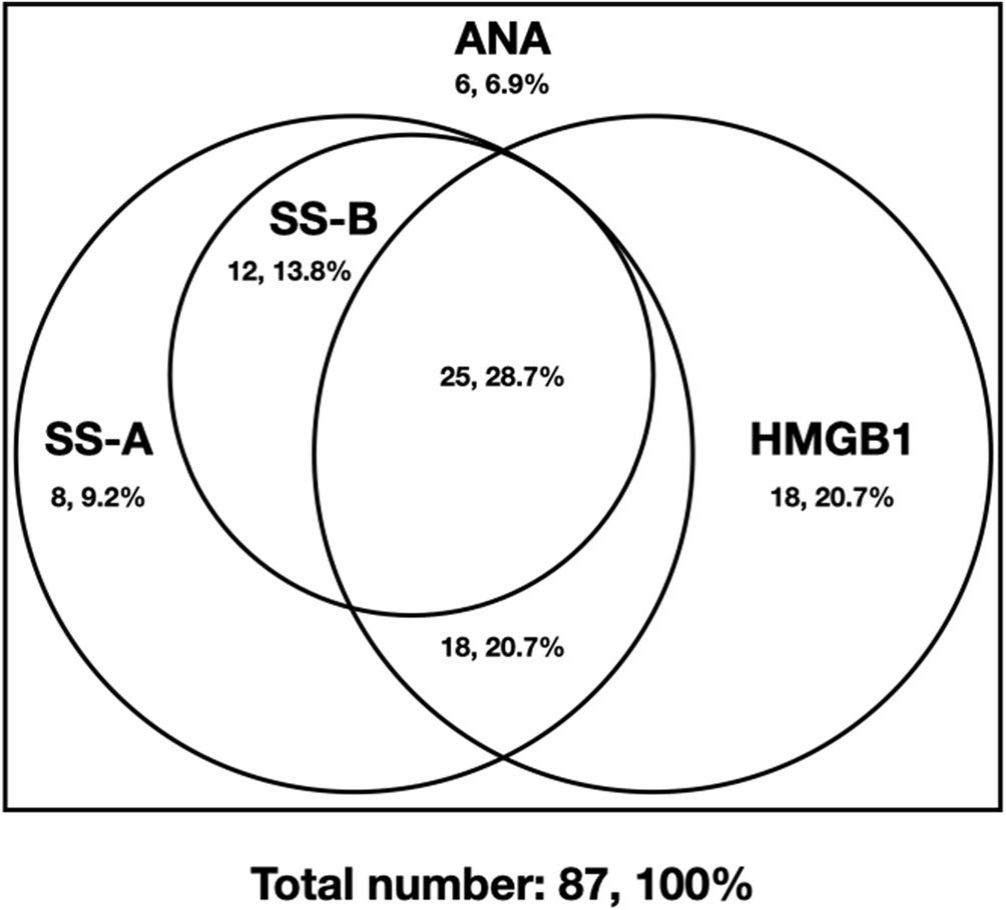
The overlap of anti-HMGB1, anti-SS-A, and anti-SS-B in ANA-IF positive SS patients.

In the SS group, 87 from 93 SS patients were positive for ANA-IF, appearing the nuclear fine speckled (AC-4) pattern with or without other fluorescence modes. The results of immunoblotting detecting anti-SS-A, anti-SS B, and anti-HMGB1 antibodies were shown in Fig. 2. Sixty-one patients (70.1%) were positive for anti-HMGB1, among whom: 25 patients (28.7%) were also detected anti-SS-A and anti-SS-B, 18 patients (20.7%) were also detected anti-SS-A, and 18 patients (20.7%) were only detected anti-HMGB1 positive. Among those patients with negative anti-HMGB1 (26, 29.9%), 20 patients (23.0%) were detected anti-SS-A antibodies, 12 patients (13.8%) were detected anti-SS-B antibodies, and 6 patients (6.9%) were detected none.

### The anti-HMGB1 antibody showed a typical nuclear fine speckled (AC-4) pattern

**Figure 3:**
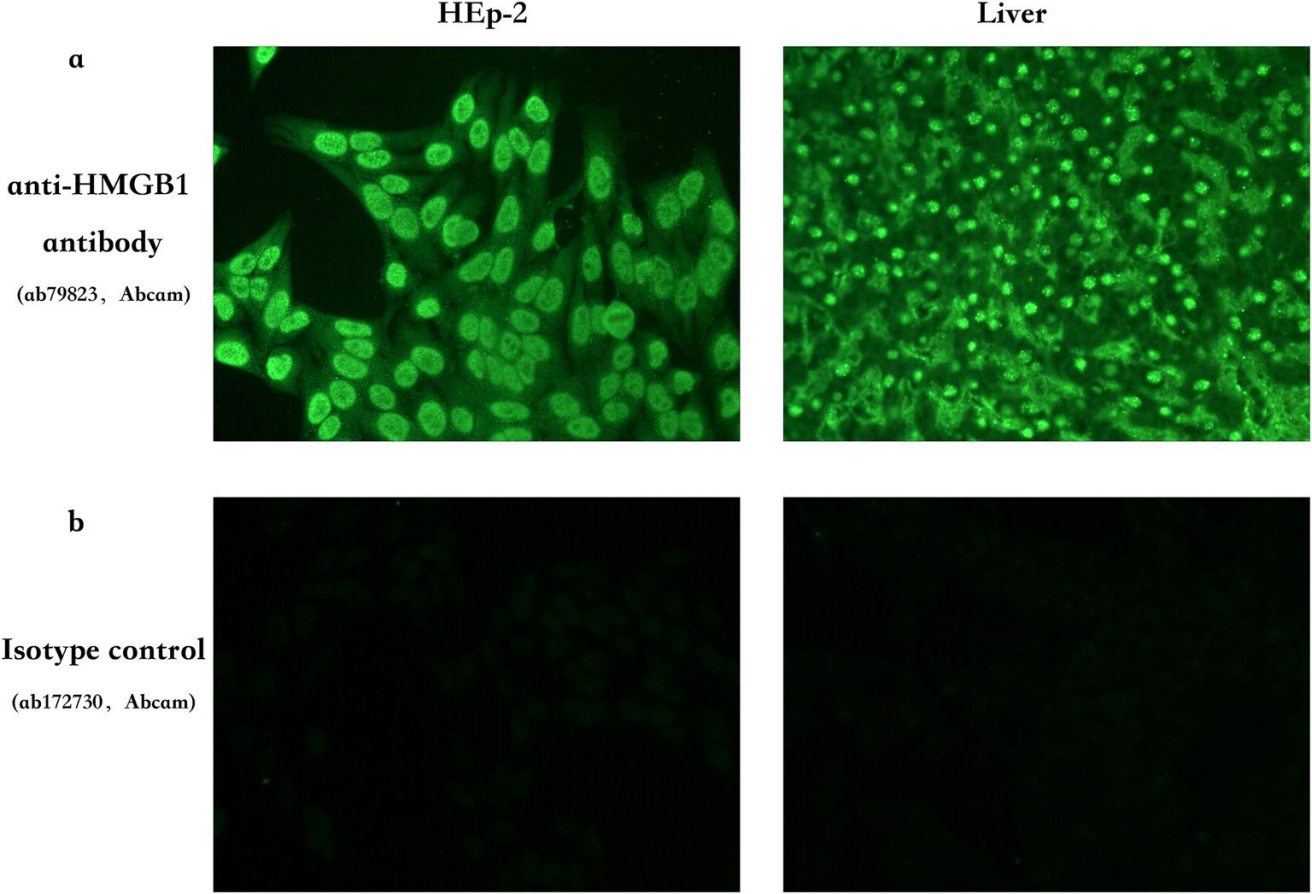
Fluorescence pattern of anti-HMGB1 antibody detected with IIFT. (**a**) Commercial anti-HMGB1 antibody was used to determine the location of HMGB1 and the typical fluorescence pattern in fixed HEp-2 cells and monkey liver slice. (**b**) Isotype control antibody was used to rule out the nonspecific fluorescence.

In the 24 ANA-IF positive cases without the detections of SS-A and SS-B, 18 cases were detected positive for anti-HMGB1 antibody. Commercial anti-HMGB1 antibody was used to further study the fluorescence pattern of anti-HMGB1 antibody. The test took fixed HEp-2 cells and monkey liver slices as the detection matrix. The test results were shown in Fig. 3. The ANA pattern of anti-HMGB1 antibody presented the nuclear fine speckled (AC-4) as found in SS.

### Some ANA-IF positive in SS patients came from anti-HMGB1 autoantibodies was confirmed by using HMGB1 knockout cell line B16^HMGB1−^

**Figure 4:**
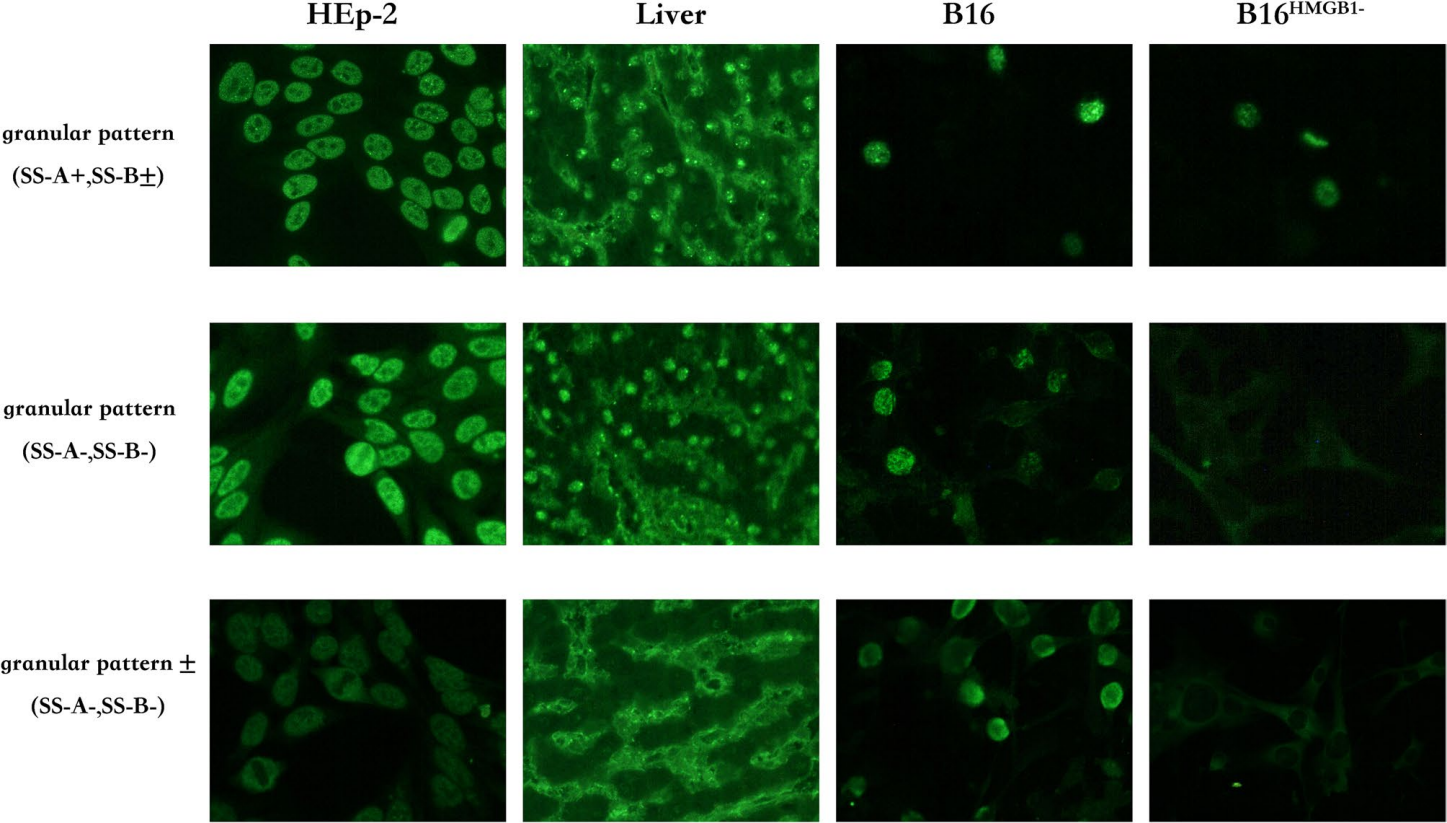
ANA-IF test with HMGB1 knockout cells in SS patients. HMGB1 knockout and wildtype cells were pre-fixed on slides as the matrix to study the anti-HMGB1 antibody’s contribution to ANA-IF, especially in ANA-IF positive, SS-A and SS-B negative patients. Three representative results were shown, Upper: ANA (+), (SS-A+, SS-B±), there are nuclear fine speckled (AC-4) pattern in both B16 and B16^HMGB1−^ cells. Middle: ANA (+), (SS-A−, SS-B−), there is nuclear fine speckled (AC-4) pattern in B16 cells, while no fluorescence in B16^HMGB1−^ cells. Down: ANA (±), (SS-A−, SS-B−), there is nuclear fine speckled (AC-4) pattern in B16 cells, while no fluorescence in B16^HMGB1−^ cells.

In order to further investigate the existence of anti-HMGB1 antibodies in SS, we constructed a Cas9-induced HMGB1 knockout B16 cell line (B16^HMGB1−^) in our lab (see supplementary methods, and Suppl Fig. 2). Taking the wild type mouse melanoma cell line (B16) and HMGB1 knockout type B16^HMGB1−^ as the detection matrix, the serum of the SS patients with ANA-IF presented nuclear fine speckled (AC-4) pattern reran the IIFT. The detection results were shown in Fig. 4. ANA(+), SS-A(+), and SS-B(±) serum still showed nuclear fine speckled (AC-4) pattern in B16^HMGB1−^; while ANA (+ or ±), SS-A (−), and SS-B (−) serum presented nuclear fine speckled (AC-4) pattern in wild type B16, and there was no fluorescence in B16^HMGB1−^, which confirmed that ANA-IF fluorescence came from the anti-HMGB1 autoantibodies in this patient.

## Discussion

HMGB1 is a highly conserved non-histone nuclear protein that is abundantly expressed in most eukaryotic cells (10^6^ molecules/cell). Composed of 215 amino acids, HMGB1 in the nucleus can regulate the stability of nucleosomes, participate in the recombination, replication, repair, and transcription of DNA, and can be actively or passively released under physiological and pathological conditions to exert an inflammatory cytokine-like role. In the nucleus, the HMGB1 protein generally binds to the minor groove of the DNA double helix on the nucleosome, and the nucleosome and its components, such as nucleosomes, histones, DNA, etc., are a large source of autoantigens^10,11^. Based on these, it is inferred that HMGB1 may also cause an autoimmune response, produce autoantibodies, and cause autoimmune diseases. A large number of studies, including our previous basic work, have shown that HMGB1 plays a role in the occurrence and development of infectious diseases and autoimmune diseases, researchers found serum HMGB1 levels are increased in SS patients and more specifically in the patients with SSA autoantibodies^12^. As an alarmin, HMGB1 is also a potential therapy target in SS, and researchers found that suppression of HMGB1 ameliorates SS-triggered xerostomia in a mouse model^13^. It, therefore, is an important point in the regulation of immune homeostasis, which may be an important target for the diagnosis and treatment of related diseases.

Laboratory indicators play a key role in the diagnosis and efficacy monitoring of autoimmune diseases. Indirect fluorescent ANA screening and detection of related autoantibodies can provide important evidence for the diagnosis and differential diagnosis of autoimmune diseases^1^. In SS, ANA-IF normally shows a nuclear fine speckled (AC-4) pattern. The presence of multiple autoantibodies in patients with SS is one of the characteristics of the disease. Among these patients, anti-SS-A autoantibody can be detected in about 70% of the patients, and SS-B autoantibody can be detected in 45% of the patients. In some ANA-positive patients, both anti-SS-A and SS-B antibodies are negative, indicating that there are some other autoantibodies with other characteristics in SS patients. Through in-house developed ELISA and immunoblotting, we found that the serum anti-HMGB1 antibody level was higher in SS patients than in healthy controls. Taking 8 RU/ml as the cut-off value, the sensitivity and specificity of diagnosing SS reached 64.5% and 96.9%, respectively, and the area under the ROC curve was 0.83, which had a potential clinical application value.

The nuclear components are abnormally recognized by the body's immune system and produce autoantibodies, which is important pathogenesis of autoimmune diseases. Whether HMGB1, as a non-histone nuclear protein, can cause the body's autoimmune response and lead to pathological changes is one of the hotspots of research. Many studies have shown that anti-HMGB1 antibodies can be detected in patients with systemic lupus erythematosus (SLE)^14–17^, and it is related to the severity of the disease. A variety of autoantibodies can appear in patients with autoimmune diseases, which have certain disease specificities, such as anti-ds-DNA antibodies and anti-Sm antibodies in SLE patients. There are also some antibodies that can be presented in multiple autoimmune diseases, for example, anti-ss-DNA (single-stranded DNA) antibodies can be detected in SLE, mixed connective tissue disease (MCTD), and polymyositis/dermatomyositis.

In this study, it was found through immunoblotting detection that 61 (70.1%) of 87 patients with ANA-IF exhibit a typical nuclear fine speckled (AC-4) pattern were positive for anti-HMGB1, among whom 43 patients (49.4%) were positive for anti-SS-A, and 20.7% (18) were positive for anti-HMGB1 alone, suggesting that anti-HMGB1 antibody was one of the sources of fluorescence in the ANA-IF experiment. Then commercial anti-HMGB1 antibody was used to study the fluorescence pattern of the anti-HMGB1 antibody. Fixed HEp-2 and monkey liver slices were used as the matrix, and the anti-HMGB1 antibody presented a typical nuclear fine speckled (AC-4) pattern, which was consistent with our hypothesis. Some studies have shown that the anti-HMGB1 antibody presented a diffuse cytoplasmic staining pattern, which is believed to be caused by activation of the matrix cells, and then HMGB1 exits the nucleus and enters the cytoplasm^16^. Our experimental results showed that the fluorescence pattern of the anti-HMGB1 antibody in HEp-2 showed a nuclear fine speckled (AC-4) pattern, and the fluorescence in the cytoplasm was very weak. The fluorescence in the monkey liver slices also consists of the findings in HEp-2 cells. Taking the fact that HMGB1 is a nuclear protein into consideration, we believe that the nuclear fine speckled (AC-4) pattern is the actual pattern for anti-HMGB1 antibody.

In order to further confirm that the anti-HMGB1 antibody is one of the characteristic autoantibodies of SS, we used the Cas9 technique to construct an HMGB1 knockout mouse melanoma cell line B16^HMGB1−^ to repeat the ANA-IF experiment. The results showed that ANA (+ or ±), SS-A (−), and SS-B (−) serums showed nuclear fine speckled (AC-4) pattern in wild-type B16, but there was no fluorescence in B16^HMGB1−^, confirming that ANA-IF fluorescence may come from the anti-HMGB1 autoantibodies in some patients.

In conclusion, the results of this study show that the anti-HMGB1 antibody may be one of the characteristic autoantibodies of SS. The detection of anti-HMGB1 antibody can provide laboratory evidence for clinical diagnosis of SS, and further improve the accuracy of SS diagnosis based on the existing detection indicators. Further research on the role of HMGB1 in the pathogenesis of SS may lead to the development of new drugs or treatment strategies for the treatment of SS.

## Supplementary Information

Supplementary Information.

## Abbreviations

SS: Sjögren's syndrome
HMGB1: High mobility group box 1
ANA: Antinuclear antibodies
IIFT: Indirect immunofluorescent antibody test
