# Supplementary material for: Anti-HMGB1 antibody is a potential characteristic autoantibody for Sjögren's syndrome
Source: Sci Rep. 2022 Apr 11;12:6020. doi: 10.1038/s41598-022-10007-3 (PMC9001690; doi:10.1038/s41598-022-10007-3)
Supplement: Supplementary file 1 — Supplementary Information. [file 41598_2022_10007_MOESM1_ESM.pdf]

Supplementary methods:

CONSTRUCTION OF HMGB1 KNOCKOUT CELL LINE ($B16^{HMGB1-}$) IN MOUSE MELANOMA CELL LINES B16

Guide RNA, proper targets, and primers were designed with the software provide by Feng Zhang's Lab (http://crispr.mit.edu/). Plasmid pX330 (Addgene) with the gRNA (pX330-HMGB1) were transfected into B16 cells. Puromycin was used to select the cells transfected with the plasmids, and serial dilutions was used to isolate the clonal cell line. The successful construction of HMGB1 knockout cell line ($B16^{HMGB1-}$) was confirmed by sequence of the PCR products targeting the ATG gene area of HMGB1 and Western blotting.

SupplTable 1: The gRNAs and primers used to generate HMGB1 knockout B16 cells

**gRNA and primers**	**Sequences**
gRNA-1F	CACCGGGAGATCCTAAAAAGCCGAG
gRNA-1R	AAACCTCGGCTTTTTAGGATCTCCC
gRNA-2F	CACCGATTTTGCCTCTCGGCTTTTT
gRNA-2R	AAACAAAAAGCCGAGAGGCAAAATC
HMGB1-F	ACCGCTTCTAGGAATTGGCT
HMGB1-R	GTGCATTGGGGTCCTTGAAC

Supplementary figure legends

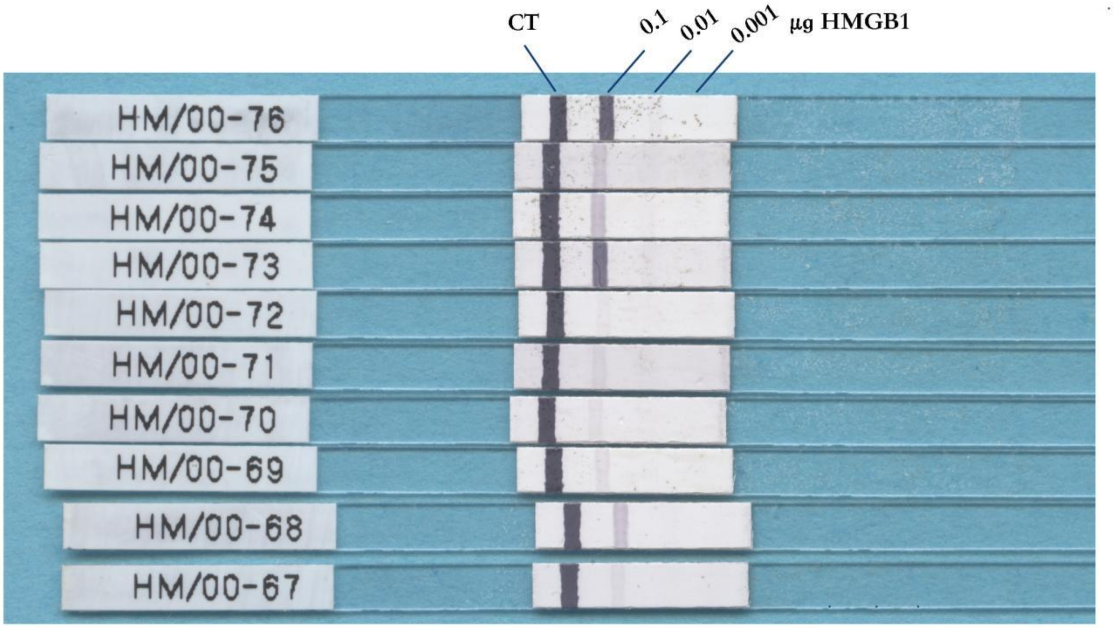

SupplFig. 1. Detection of anti-HMGB1 antibodies in SS with immunoblotting

Indirect immunoblotting was also used to confirm the detection of anti-HMGB1 antibodies in SS.

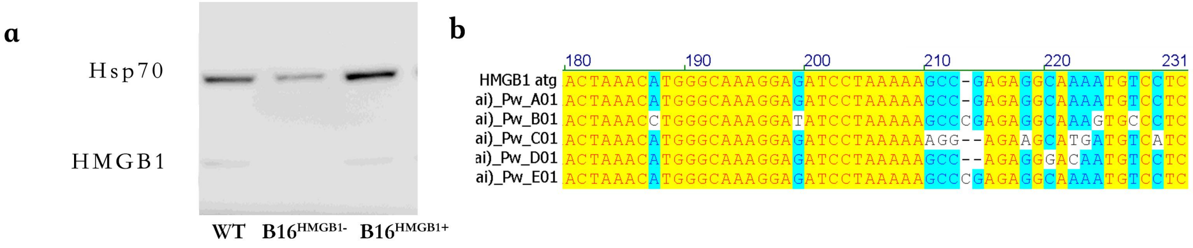

SupplFig. 2. The construction of B16^{HMGB1-} was confirmed with Western blotting and sequence

a) Western blotting results showed that no HMGB1 protein was detected in B16^{HMGB1-} cells. b) Sequence of the PCR products showed Insertion and deletion mutations in

B16^{HMGB1-} cells.

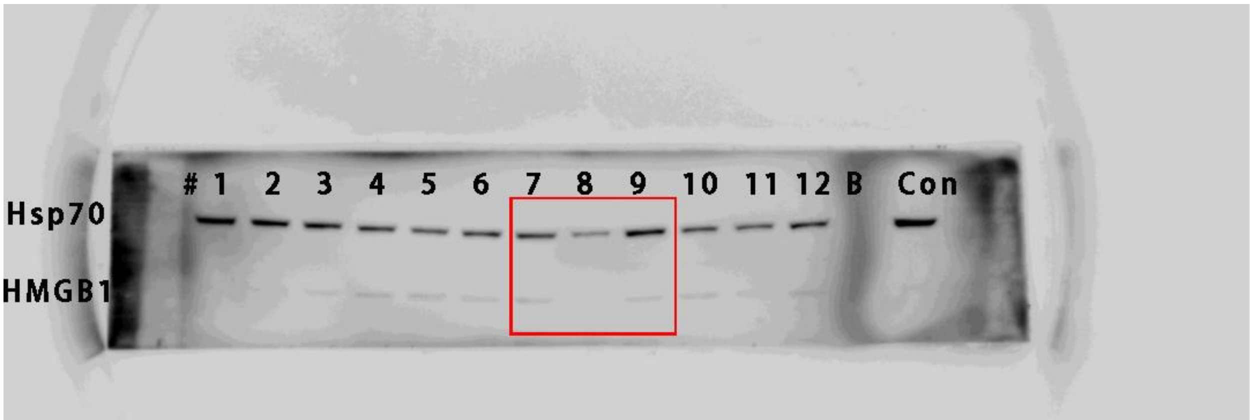

The original image of supplFig. 2a
